# The association of *AGO1* (rs595961G>A, rs636832A>G) and *AGO2* (rs11996715C>A, rs2292779C>G, rs4961280C>A) polymorphisms and risk of recurrent implantation failure

**DOI:** 10.1042/BSR20190342

**Published:** 2019-11-26

**Authors:** Chang Soo Ryu, Young Ran Kim, Jung Oh Kim, Hui Jeong An, Sung Hwan Cho, Eun Hee Ahn, Ji Hyang Kim, Woo Sik Lee, Nam Keun Kim

**Affiliations:** 1 Department of Biomedical Science, College of Life Science, CHA University, 335 Pangyo-ro, Bundang-gu, Seongnam 13488, South Korea; 2 Department of Obstetrics and Gynecology, CHA Bundang Medical Center, CHA University, Seongnam 13496, South Korea; 3 Fertility Center of CHA Gangnam Medical Center, CHA University, Gangnam 06135, South Korea

**Keywords:** Argonaute, miRNA, recurrent implantation failure, RISC

## Abstract

Recurrent implantation failure (RIF) is a common reproductive clinical condition treated by fertility specialists at *in vitro* fertilization (IVF) clinics. Several factors affect embryo implantation including the age of the female, the quality of embryos and the sperm, genetics, immunologic factors. Here, we investigated the association of Argonaute 1 (*AGO1*) and Argonaute 2 (*AGO2*) polymorphisms and RIF. We collected blood samples from 167 patients with RIF and 211 controls. Genetic polymorphisms were detected by polymerase chain reaction (PCR) – restriction fragment length polymorphism analysis and real-time PCR. We found that the *AGO2* rs4961280C>A polymorphism (adjusted odds ratio [AOR] = 1.984; *P* = 0.023) was significantly associated with RIF. Furthermore, in RIF patients with three or more consecutive implantation failure, the *AGO2* rs4961280C>A CA genotype (AOR = 2.133; *P* = 0.013) and dominant model (AOR = 2.272; *P* = 0.006) were both significantly associated with prevalence of RIF. An analysis of variance revealed that patients with the *AGO2* rs2292779C>G genotypes (CC: 6.52 ± 2.55; CG: 7.46 ± 3.02; GG: 8.42 ± 2.74; *P* = 0.044) and the dominant model (CC: 6.52 ± 2.55; CG+GG: 7.70 ± 2.97; *P* = 0.029) exhibited significantly increased white blood cell levels. Furthermore, patients with the *AGO1* rs595961G>A dominant model (GG: 36.81 ± 8.69; GA+AA: 31.58 ± 9.17; *P* = 0.006) and the *AGO2* rs4961280C>A recessive model (CC+CA: 35.42 ± 8.77; AA: 22.00 ± 4.24; *P* = 0.035) exhibited a significantly decreased number of CD4^+^ helper T cells. Our study showed that *AGO1* and *AGO2* polymorphisms are associated with the prevalence of RIF. Hence, the results suggest that variations in *AGO1* and *AGO2* genotypes may be useful clinical biomarkers for the development and prognosis of RIF.

## Introduction

Human reproduction is a relatively inefficient process, with only a 25% chance that sperm will fertilize an oocyte during a single menstrual cycle and result in pregnancy [1]. Recurrent implantation failure (RIF) is one of the most common reproductive clinical conditions treated by fertility specialists at *in vitro* fertilization (IVF) clinics [2]. Furthermore, RIF is defined as the clinical symptom that implanted embryo was undergone two or more reiterated failures before reaching the recognize stage [3]. Additionally, RIF was defined as the failure to achieve pregnancy following two or more completed *in vitro* fertilization-embryo transfer (IVF-ET) cycles with one or two good quality embryos [4]. Unfortunately, several different definitions are used to describe RIF, making it difficult to precisely identify affected patients. Many factors interfere with successful embryo implantation and contribute to RIF, including the age of the female, the indication for IVF, the treatment procedure, the quality and number of embryos, the quality of the sperm, thrombophilia, genetics, immunologic factors, and hormonal control of endometrial receptivity [5–7]. Thus, successful implantation is a complex process that is affected by both paternal and maternal factors. [8]. During a short period of 7 to 10 days in the secretory phase of a normal menstrual cycle, implantation can occur when a fertilized embryo develops into a blastocyst as it migrates to the uterus and successfully attaches to the uterine lining [9].

MicroRNAs (miRNA) are thought to play key roles in evolutionary processes, especially in the development of embryo complexity [10]. Over the past 7–10 years, the majority of miRNA-related research has compared cancer cells and normal cells [11]. However, the researches on the regulatory roles of miRNAs in physiological process such as pregnancy are increasing [12,13]. Furthermore, miRNAs are well-known biologic regulators of cell cycle progression, proliferation, and differentiation that occur in the endometrium during the menstrual cycle [14,15]. The relevance of these roles has recently been linked to findings that demonstrated a role for miRNAs in down-regulating the expression of certain cell cycle genes in secretory-phase endometrial epithelium [16,17]. In addition, aberrant miRNA expression can have serious consequences and is already associated with human reproductive disorders such as endometriosis and recurrent pregnancy loss [18]. These miRNAs are transcribed from DNA as longer sequences known as pri-miRNAs and pre-miRNAs. In the cytoplasm, mature miRNAs form the RNA-induced silencing complex (RISC) with Argonaute (Ago) proteins and its function was known to inhibiting protein translation. [19]. Furthermore, the RISC was always formed from the Ago proteins, either with Ago or PIWIs during the assembly process including the several steps (e.g. loading and maturation) [20].

Proteins in the Argonaute family, which are known to the functional core of RISC, were divided into AGOs (Ago1, Ago2 of flies and Ago1, Ago2, Ago3, Ago4 of mammals) that bind the miRNAs and siRNAs, and PIWIs that bind the piRNAs [21]. The four mammalian argonaute genes encodes the same domain structure found in all Argonaute proteins, including four primary domains (N, PAZ, MID, and PIWI) and two linker regions L1 and L2 [22]. In contrast, prokaryotic Argonaute protein, *pAgo1*, contains several insertions that affected the primary domain and L2 linker of Argonaute, indicating possible biological roles following potential interactions with guide or target RNA molecules or other proteins [23]. In mammalian, the proteins of Argonaute family have identified in purified RISC and played a critical role between phosphorylated siRNA duplexes and RISC reconstitutions at the RNAi pathway [24]. Furthermore, the human Ago2 has a potential function in guide binding and target RNA recognition, as well as in the recruitment of Ago2-associated protein factors [25]. More specifically, nucleotides 2–6 of the guide RNA are defined as the “seed sequence” and support the “seed-paring” model of miRNA targeting that proposes Argonaute pre-arranges miRNA nucleotides 2–7 in an A-form configuration [26]. These conformational changes have been linked to the importance of pairing to nucleotide 7 for effective miRNA targeting and are possibly used to monitor the recognition of miRNA target sites [27]. Several endogenous miRNAs and their RISCs have been genetically programmed to regulate gene expression and have important roles in the growth and development of an organism [28]. Notably, the Argonaute proteins are important components of RISCs, and these RISCs that were consisting of miRNAs and Argonaute proteins regulate gene expression, affecting cell growth and the development of organisms [29]. Furthermore, the embryonic stem cell without argonaute 2 is delayed in self-renewal and differentiation [30]. Unfortunately, the Argonaute protein studies of affecting implantation progress were mainly reported in the mouse model and had not many reported in the human groups [31–33]. Therefore, the potential exists that the Argonaute subfamily genes are associated with risk of RIF in the human group study.

In the present study, we utilized a case–control study to investigate the role of *AGO1* and *AGO2* gene polymorphisms (*AGO1* rs595961G>A, *AGO1* rs636832A>G, *AGO2* rs11996715C>A, *AGO2* rs2292779C>G, and *AGO2* rs4961280C>A) in RIF patients and controls of the Korean population. Furthermore, we were chosen *AGO1* and *AGO2* gene polymorphisms because the argonaute gene polymorphisms were already reported in other conditions and diseases but had not been reported in RIF [34–38]. To our knowledge, this is the first study to provide evidence of the role of the *AGO1* and *AGO2* polymorphisms in RIF in Korean individuals.

## Materials and methods

### Study population

All the study participants were recruited from the CHA Bundang Medical Center Department of Obstetrics and Gynecology, CHA University (Seongnam, Korea) between March 2010 and December 2012. In the present study, RIF was defined as the failure to achieve pregnancy following two or more completed fresh IVF-ET cycles with one or two good quality embryos. Each transferred embryo was cleaved into more than 10 cells. Blood samples were collected from 167 patients with RIF and 211 controls. Serum hCG concentrations were less than 5 mIU/ml 14 days after embryo transfer. All transferred embryos were examined by the embryologist before transfer and were considered to be of good quality. The study was approved by the Institutional Review Board of CHA Bundang Medical Center (reference no.PBC09-120) and all patients provided written informed consent. All study protocols followed the recommendations of the Declaration of Helsinki.

The exclusion criteria for a diagnosis of RIF generally includes implantation failures due to anatomic, chromosomal, hormonal, infectious, autoimmune, or thrombotic defects or disorders. Accordingly, subjects who were diagnosed with RIF due to these causes were excluded from the study group. The participants of smoking status were excluded from the RIF patients and control groups. Anatomical abnormalities were evaluated using several imaging methods, including sonography, hysterosalpingogram, hysteroscopy, computerized tomography, and magnetic resonance imaging. Karyotyping was performed using standard protocols. Hormonal abnormalities triggered by causes such as hyperprolactinemia, luteal insufficiency, and thyroid disease was excluded by measuring prolactin, thyroid-stimulating hormone, free T4, follicle-stimulating hormone (FSH), luteinizing hormone (LH), and progesterone levels in peripheral blood. Lupus anticoagulant and anticardiolipin antibodies were measured to exclude patients with autoimmune disorders such as lupus or antiphospholipid syndrome. Thrombotic disease was defined as thrombophilia and was evaluated by assessing protein C and protein S deficiencies and by the presence of the anti-α2 glycoprotein antibody. Semen analysis, karyotyping, and hormonal assays, including estradiol, testosterone, FSH, and LH were performed for male partners. Of the initial 215 patients screened, 48 subjects were excluded due to hypothyroidism, trisomy, intrauterine adhesion, chromosomal disorders, or antiphospholipid syndrome, resulting in a study population of 167 patients. Enrollment criteria for the control group included regular menstrual cycles, normal karyotype (46, XX), a history of at least one natural pregnancy, and no history of pregnancy loss. Data were collected in the same manner for both groups.

### Genotyping

DNA samples were extracted from blood samples collected from the RIF patients and controls using a G-DEX(TM) Genomic DNA Extraction Kit for blood (iNtRON Biotechnology, Seongnam, South Korea). The classification of alleles of the genetic polymorphism was confirmed to the East Asian population on the 1000 Genomes study. Most of the genetic polymorphisms (*AGO1* rs595961G>A, *AGO1* rs636832G>A, *AGO2* rs22927779C>G, *AGO2* rs4961280C>A) were detected by polymerase chain reaction–restriction fragment length polymorphism (PCR-RFLP) and one of the genetic polymorphisms (*AGO2* rs11996715C>A) was detected by real-time PCR [39,40]. *AGO1* rs595961G>A, *AGO1* rs636832G>A, *AGO2* rs2292779C>G, and *AGO2* rs4961280C>A were confirmed by digestion with the restriction enzymes (New England Bio Laboratories, Ipswich, MA, U.S.A.) at 37°C for 16 h. The detailed information of PCR primers, restriction enzymes, and size of fragments after enzymatic cleavage was showed Supplementary Table S1. Furthermore, each genotype was confirmed by electrophoretic separation on 4% agarose gels. For each Argonaute polymorphism, 30% of the PCR products were randomly chosen for a duplicate PCR assay and confirmed by DNA sequencing to validate the RFLP findings. DNA sequencing was performed using an ABI 3730xl DNA Analyzer (Applied Biosystems, Foster City, CA, U.S.A.) and the concurrence of the quality of each sample was 100%.

### Statistical analysis

Differences in the genotype frequencies of the polymorphisms were compared between the RIF patients and control subjects using a Fisher’s exact test and logistic regression. The odds ratio (OR) and 95% confidence interval (CI) were used as a measure of the strength of the association between the genotype frequencies and RIF. The subtype analyses of RIF were performed between stratified groups from implantation failure number. The OR and 95% CI were also used to assess the relationship between each specific polymorphism and allele combination. The associations between the polymorphisms and RIF incidence were calculated using adjusted ORs (AORs) and 95% CIs from logistic regression adjusted for age. Differences resulting in a *P* <0.05 were considered statistically significant. A false discovery rate (FDR) correction was performed to adjust for multiple comparisons. All the polymorphisms were in Hardy–Weinberg equilibrium (*P*>0.05). Statistical analyses were performed using GraphPad Prism 4.0 (GraphPad Software, Inc., San Diego, CA, U.S.A.), StatsDirect statistics software version 2.4.4 (StatsDirect Ltd., Altrincham, U.K.), HaploView 4.1 (Broad Institute of MIT and Harvard, Boston, MA, U.S.A.), and HAPSTAT 3.0 (University of North Carolina, Chapel Hill, NC, U.S.A.). Gene–gene interaction analysis was performed using the open source multidimensional reduction (MDR) software package v.2.0 (www.epistasis.org). Furthermore, all possible combinations of the polymorphisms were studied to determine the combinations with strong synergistic effects.

## Results

### The baseline characteristics

The demographic characteristics and clinical variables of RIF patients and control subjects are shown in Table 1. The mean ages of the RIF patients and control subjects were 34.67 ± 3.65 and 34.27 ± 5.48 years, respectively. The prothrombin time (PT) values, activated partial thromboplastin time (aPTT) values and total cholesterol values were significantly lower in the RIF patient group than in the control group. Furthermore, the estrogen (E2) values, luteinizing hormone (LH) values and fasting blood sugar (FBS) were significantly higher in the RIF patient group than in the control group.

**Table 1:** Baseline characteristics between controls and RIF patients

Characteristic	Controls (*n* = 211)	RIF patients (*n* = 167)	*P*^1^
Age (years, mean ± SD)	34.27 ± 5.48	34.67 ± 3.65	0.423
BMI (kg/m^2^, mean ± SD)	21.78 ± 3.53	21.00 ± 2.84	0.215^2^
PLT (10^3^/μl, mean ± SD)	240.62 ± 67.01	239.59 ± 60.47	0.888
PT (s, mean ± SD)	11.63 ± 3.37	10.82 ± 2.18	0.024^2^
aPTT (s, mean ± SD)	33.43 ± 3.70	29.32 ± 3.39	<0.0001
FSH (mIU/ml, mean ± SD)	8.15 ± 2.84	8.98 ± 4.96	0.611^2^
LH (mIU/ml, mean ± SD)	3.26 ± 1.77	4.88 ± 2.39	<0.0001^2^
E2 (pg/ml, mean ± SD)	26.19 ± 14.68	71.30 ± 187.92	<0.0001^2^
FBS (mg/dl, mean ± SD)	91.81 ± 15.60	99.49 ± 10.13	0.0004^2^
Total cholesterol (mg/dl, mean ± SD)	228.47± 62.21	176.40 ± 25.86	<0.0001^2^

Abbreviations: SD, standard deviation; PLT, platelet; PT, prothrombin time; aPTT, activated partial thromboplastin time; FSH, follicle stimulating hormone; LH, luteinizing hormone; E2, estradiol; FBS, fasting blood sugar.

1 *P*-values were calculated by two-sided *t*-test for continuous variables and chi-square test for categorical variables.

2 Mann–Whitney test for continuous data.

### Genotype frequencies of the *AGO1* and *AGO2* gene polymorphisms between RIF patients and controls

We investigated the *AGO1* rs595961G>A, *AGO1* rs636832A>G, *AGO2* rs11996715C>A, *AGO2* rs2292779C>G and *AGO2* rs4961280C>A polymorphisms between RIF patients including the numbers of times of RIF (RIF ≥ 2 and RIF ≥ 3) and control groups (Table 2). We calculated the AOR using logistic regression analyses with respect to age. The *AGO1* and *AGO2* polymorphism frequencies were all in Hardy–Weinberg equilibrium (*P* > 0.05). Furthermore, we detected several different associations between RIF patients and controls in the genotype frequency analysis. The *AGO2* rs4961280C>A CA genotype and the dominant model (CC vs. CA+AA) were significantly associated with increased RIF prevalence (Table 2). Furthermore, in analysis of numbers of RIF, we detected associations between *AGO2* polymorphisms and number of RIF occurrences. Specifically, RIF ≥ 3 was significantly associated with the *AGO2* rs4961280C>A polymorphism (Table 2).

**Table 2:** Genotype frequency of Argonaute gene polymorphisms between RIF patients and controls

Genotypes	Controls (*n* = 211)	RIF ≥ 2 (*n* = 167)	AOR (95% CI)^1^	*P*	RIF ≥ 3 (*n* = 153)	AOR (95% CI)^1^	*P*
*AGO1* rs595961G>A							
GG	163 (77.3)	123 (73.7)	1.000 (reference)		112 (73.2)	1.000 (reference)	
GA	44 (20.9)	41 (24.6)	1.251 (0.769–2.036)	0.367	38 (24.8)	1.282 (0.779–2.109)	0.329
AA	4 (1.9)	3 (1.8)	0.982 (0.216–4.469)	0.981	3 (2.0)	1.073 (0.235–4.894)	0.927
Dominant (GG vs. GA+AA)			1.228 (0.766–1.969)	0.395		1.263 (0.780–2.047)	0.343
Recessive (GG+GA vs. AA)			0.930 (0.205–4.219)	0.926		1.010 (0.223–4.587)	0.989
HWE *P*	0.673	0.844					
*AGO1* rs636832A>G							
AA	105 (49.8)	95 (56.9)	1.000 (reference)		89 (58.2)	1.000 (reference)	
AG	95 (45.0)	62 (37.1)	0.730 (0.476–1.117)	0.147	54 (35.3)	0.685 (0.441–1.065)	0.093
GG	11 (5.2)	10 (6.0)	0.999 (0.405–2.461)	0.998	10 (6.5)	1.059 (0.429–2.614)	0.901
Dominant (AA vs. AG+GG)			0.760 (0.504–1.145)	0.189		0.727 (0.477–1.109)	0.139
Recessive (AA+AG vs. GG)			1.130 (0.467–2.736)	0.786		1.228 (0.506–2.977)	0.650
HWE *P*	0.073	0.978					
*AGO2* rs11996715C>A							
CC	103 (48.8)	71 (42.5)	1.000 (reference)		67 (43.8)	1.000 (reference)	
CA	82 (38.9)	78 (46.7)	1.393 (0.902–2.150)	0.135	71 (46.4)	1.347 (0.865–2.100)	0.188
AA	26 (12.3)	18 (10.8)	0.997 (0.508–1.957)	0.994	15 (9.8)	0.879 (0.433–1.783)	0.721
Dominant (CC vs. CA+AA)			1.297 (0.862–1.953)	0.213		1.232 (0.810–1.874)	0.329
Recessive (CC+CA vs. AA)			0.844 (0.445–1.602)	0.604		0.754 (0.384–1.481)	0.412
HWE *P*	0.133	0.616					
*AGO2* rs2292779C>G							
CC	79 (37.4)	69 (41.3)	1.000 (reference)		65 (42.5)	1.000 (reference)	
CG	109 (51.7)	70 (41.9)	0.727 (0.467–1.132)	0.158	64 (41.8)	0.702 (0.446–1.104)	0.125
GG	23 (10.9)	28 (16.8)	1.388 (0.732–2.632)	0.316	24 (15.7)	1.255 (0.648–2.431)	0.500
Dominant (CC vs. CG+GG)			0.841 (0.554–1.275)	0.414		0.797 (0.520–1.221)	0.297
Recessive (CC+CG vs. GG)			1.641 (0.906–2.973)	0.102		1.513 (0.818–2.800)	0.187
HWE *P*	0.105	0.163					
*AGO2* rs4961280C>A							
CC	189 (89.6)	134 (80.2)	1.000 (reference)		121 (79.1)	1.000 (reference)	
CA	22 (10.4)	31 (18.6)	1.984 (1.100–3.578)	0.023	30 (19.6)	2.133 (1.175–3.872)	0.013
AA	0 (0.0)	2 (1.2)	NA	0.998	2 (1.3)	NA	0.998
Dominant (CC vs. CA+AA)			2.110 (1.177–3.781)	0.012		2.272 (1.260–4.097)	0.006
Recessive (CC+CA vs. AA)			NA	0.998		NA	0.998
HWE *P*	0.424	0.891					

Abbreviations: RIF, recurrent implantation failure; AOR, adjusted odds ratio; AGO, argonaute; HWE, Hardy–Weinberg equilibrium; 95% CI, 95% confidence interval; NA, not applicable; ^1^Adjusted by age.

### Analyses of the *AGO1* and *AGO2* gene polymorphism allele combinations between RIF patients and controls

Next, we analyzed the allele combinations and compared the RIF patients and controls (Table 3). Based on the MDR method, the G-A-C-A allele combination in the *AGO1* rs595961G>A, *AGO1* rs636832A>G, *AGO2* rs2292779C>G, and *AGO2* rs4961280C>A polymorphisms, the G-G-C-A allele combination in the *AGO1* rs595961G>A, *AGO1* rs636832A>G, *AGO2* rs2292779C>G, and *AGO2* rs4961280C>A polymorphisms, the A-G-C-C allele combination in the *AGO1* rs595961G>A, *AGO1* rs636832A>G, *AGO2* rs2292779C>G, and *AGO2* rs4961280C>A polymorphisms, the A-G-G-A allele combination in the *AGO1* rs595961G>A, *AGO1* rs636832A>G, *AGO2* rs2292779C>G, and *AGO2* rs4961280C>A polymorphisms, the A-G-C allele combination in the *AGO1* rs595961G>A, *AGO1* rs636832A>G, and *AGO2* rs2292779C>G polymorphisms were significantly associated with increased prevalence of RIF (*P* < 0.05). Conversely, the A-A-C-C allele combination in the *AGO1* rs595961G>A, *AGO1* rs636832A>G, *AGO2* rs2292779C>G, and *AGO2* rs4961280C>A polymorphisms, the G-G allele combination in the *AGO1* rs595961G>A and *AGO1* rs636832A>G polymorphisms, the A-A allele combination in the *AGO1* rs595961G>A and *AGO1* rs636832A>G polymorphisms were associated with decreased prevalence of RIF. However, the A-G allele combination in the *AGO1* rs595961G>A and *AGO1* rs636832A>G polymorphisms was associated with increased RIF prevalence.

**Table 3:** Allele combination analysis for the Argonaute gene polymorphisms in RIF patients and controls

Allele combinations	Controls (2*n* = 422)	RIF patients (2*n* = 334)	OR (95%CI)	*P*
*AGO1* rs595961G>A/*AGO1* rs636832A>G/*AGO2* rs2292779C>G/*AGO2* rs4961280C>A				
G-A-C-C	173 (41.0)	140 (41.9)	1.000 (reference)	
G-A-C-A	5 (1.2)	16 (4.7)	3.954 (1.413–11.060)	0.006
G-A-G-C	85 (20.1)	86 (25.6)	1.250 (0.861–1.817)	0.241
G-A-G-A	11 (2.7)	9 (2.6)	1.011 (0.407–2.509)	0.981
G-G-C-C	55 (13.1)	15 (4.5)	0.337 (0.183–0.622)	0.0003
G-G-C-A	0 (0.0)	4 (1.3)	11.110 (0.593–208.300)	0.042
G-G-G-C	39 (9.3)	18 (5.4)	0.570 (0.313–1.041)	0.065
G-G-G-A	2 (0.4)	0 (0.0)	0.247 (0.012–5.190)	0.505
A-A-C-C	18 (4.2)	1 (0.3)	0.069 (0.009–0.521)	0.001
A-A-C-A	2 (0.5)	0 (0.0)	0.247 (0.012–5.190)	0.505
A-A-G-C	11 (2.6)	1 (0.4)	0.112 (0.014–0.881)	0.015
A-A-G-A	0 (0.0)	0 (0.0)	NA	NA
A-G-C-C	13 (3.0)	29 (8.8)	2.757 (1.381–5.503)	0.003
A-G-C-A	2 (0.4)	2 (0.7)	1.236 (0.172–8.888)	1.000
A-G-G-C	7 (1.6)	9 (2.7)	1.589 (0.577–4.374)	0.367
A-G-G-A	0 (0.0)	4 (1.2)	11.110 (0.593–208.300)	0.042
*AGO1* rs595961G>A/*AGO1* rs636832A>G/*AGO2* rs2292779C>G				
G-A-C	180 (42.7)	154 (46.2)	1.000 (reference)	
G-A-G	94 (22.3)	96 (28.7)	1.194 (0.836–1.705)	0.330
G-G-C	54 (12.9)	20 (6.1)	0.433 (0.248–0.755)	0.003
G-G-G	41 (9.8)	17 (5.1)	0.485 (0.265–0.888)	0.017
A-A-C	19 (4.4)	1 (0.3)	0.062 (0.008–0.465)	0.0001
A-A-G	12 (2.8)	1 (0.3)	0.097 (0.013–0.758)	0.008
A-G-C	14 (3.3)	33 (9.7)	2.755 (1.422–5.337)	0.002
A-G-G	7 (1.7)	12 (3.7)	2.004 (0.770–5.217)	0.148
*AGO1* rs595961G>A/*AGO1* rs636832A>G				
G-A	274 (65.0)	250 (74.8)	1.000 (reference)	
G-G	96 (22.7)	37 (11.2)	0.422 (0.279–0.641)	<0.0001
A-A	31 (7.3)	2 (0.7)	0.071 (0.017–0.299)	<0.0001
A-G	21 (5.1)	45 (13.4)	2.349 (1.361–4.053)	0.002

Abbreviations: RIF, recurrent implantation failure; AGO, argonaute; 95% CI, 95% confidence interval; OR, odds ratio.

### Analyses of the *AGO1* and *AGO2* gene polymorphism genotype combinations between the RIF patients and controls

Next, we analyzed the genotype combinations in the RIF patients and controls (Supplementary Table S2). The results revealed that the GG/CA genotype combinations in the *AGO1* rs595961G>A and *AGO2* rs4961280C>A polymorphisms, the CC/CA genotype combinations in the *AGO2* rs11996715C>A and *AGO2* rs4961280C>A polymorphisms, and the CA/CA genotype combinations in the *AGO2* rs11996715C>A and *AGO2* rs4961280C>A polymorphisms were significantly associated with the increased prevalence of RIF (*P* < 0.05). In addition, the CC/CA genotype combinations in the *AGO2* rs2292779C>G and *AGO2* rs4961280C>A polymorphisms was associated with increased RIF prevalence. Conversely, the GG/AG genotype combinations in the *AGO1* rs595961G>A and *AGO1* rs636832A>G polymorphisms was associated with decreased RIF prevalence.

### Clinical factors in RIF patients stratified by *AGO1* and *AGO2* polymorphisms

**Figure 1:**
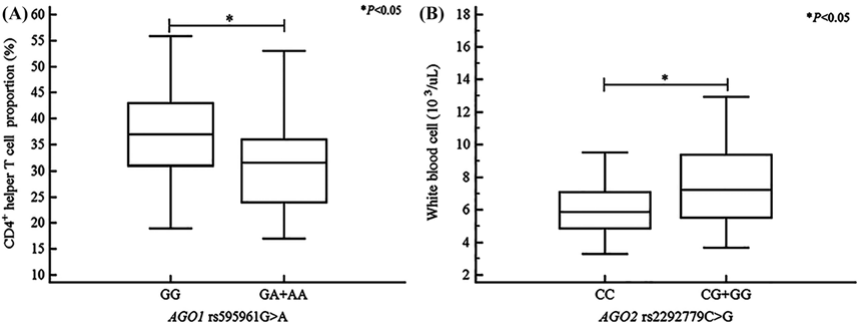
Association between differences in white blood cell levels, CD4^+^ T helper cell proportions, and the *AGO1* rs595961G>A, and *AGO2* rs2292779C>G polymorphisms in patients with recurrent implantation failure (**A**) Patients with the *AGO1* rs595961GA+AA genotype had significantly fewer CD4^+^ T helper cells than did patients with the *AGO1* rs595961GG genotype. (**B**) Patients with the *AGO2* rs2292779CG+GG genotype had significantly higher WBC levels compared with patients with the *AGO2* rs2292779CC genotype; *P < 0.05.

Using analysis of variance (ANOVA) in RIF patients, we determined that the *AGO1* rs595961G>A genotype (GG vs. GA vs. AA) and the dominant model (GG vs. GA+AA) were significantly associated with a decreased proportion of CD4^+^ helper T cells (Supplementary Table S4 and Figure 1A). The *AGO1* rs636832A>G dominant model (AA vs. AG+GG) was significantly associated with decreased CD3^+^ pan T-cell proportion (Supplementary Table S4). In addition, the *AGO2* rs22927792C>G genotype (CC vs. CG vs. GG) and the dominant model (CC vs. CG+GG) were significantly associated with increased white blood cell levels (Supplementary Table S3 and Figure 1B). Furthermore, we found that The *AGO1* rs636832A>G dominant model (AA vs. AG+GG) was associated with decreased LH levels (Supplementary Table S3). The *AGO2* rs4961280C>A genotype (CC vs. CA vs. AA) and the dominant model (CC vs. CA+AA) were associated with decreased homocysteine levels (Supplementary Table S3).

The results also indicated that the *AGO1* rs595961G>A genotype (GG vs. GA vs. AA) and the dominant model (GG vs. GA+AA) were significantly associated with activated partial thromboplastin time (Supplementary Table S3). Finally, we found that the *AGO1* rs595961G>A dominant model (GG vs. GA+AA) exhibited a tendency toward association with thyroid-stimulating hormone and CD19^+^ B cell numbers (Supplementary Table S4).

## Discussion

In the present study, we sought to determine novel markers with potential application in clinical diagnostics. To this end, we investigated the association between five polymorphisms (*AGO1* rs595961G>A, *AGO1* rs636832A>G, *AGO2* rs11996715C>A, *AGO2* rs2292779C>G, and *AGO2* rs4961280C>A) and the occurrence of RIF in a Korean population.

Previous studies identified an association between *AGO1* and *AGO2* and ovarian carcinoma [41], myeloma angiogenesis [42], as well as with an angiogenesis defect model related to inflammation [43]. Furthermore, Argonaute 1 was associated with an angiogenic pathway involving hypoxia-responsive miRNAs that could be a potentially suitable target for anti- or proangiogenesis [44]. Importantly, the regulation of Argonaute 2 is reportedly the safety mechanism that limits the range of the anti-inflammatory activity of miR-146a [45]. Our previous study also demonstrated an association between miR-146a and risk of RIF [46]. In addition, Argonaute 2 was identified as the catalytic core of mammalian RISC involved in miRNA expression, and is reportedly essential to mammalian gastrulation and mesoderm formation [47–50]. Consequently, *AGO1* and *AGO2* have previously been linked to the association with implantation due to the reported association between Argonaute and secreted miRNA in the human blastocyst, as well as human trophoblast [16,51].

In accord with our hypothesis, our analyses revealed that the *AGO2* rs4961280C>A genotypes were significantly associated with prevalence of RIF. Notably, several factors associated with RIF including white blood cell counts, FSH and LH levels, and blood urea nitrogen concentrations as well as inflammation-related factors such as CD3^+^ pan T cells, CD4^+^ helper T cells, and CD8^+^ suppressor T cells have been shown to be significantly associated with *AGO1* and *AGO2* gene polymorphisms in RIF patients. In our study, we found that all *AGO1* rs595961G>A genotypes and the dominant model were related to decreased CD4^+^ helper T cell proportions in RIF patients. The *AGO1* rs636832A>G dominant model was associated with decreased proportions of CD3^+^ pan T cell in RIF patients. Furthermore, all *AGO2* rs2292779C>G genotypes and the dominant model were associated with increased white blood cell levels in RIF patients. Finally, the *AGO2* rs4961280C>A recessive model were associated with increased CD8^+^ suppressor T-cell proportions in RIF patients. Allele frequencies of *AGO1* (rs595961G>A, rs636832A>G) and *AGO2* (rs11996715C>A, rs2292779C>G, and rs4961280C>A) polymorphisms in different world populations are presented in Table 4 [52–58]. Taken together, the results of the present study provide evidence that the *AGO1* and *AGO2* polymorphisms may play a role in RIF.

**Table 4:** Allele frequencies of *AGO1* (rs595961A>G, rs636832A>G) and *AGO2* (rs11996715C>A, rs2292779C>G, rs4961280C>A) polymorphisms in different world populations

Population	*N*	*AGO1* rs595961G>A		*AGO1* rs636832A>G		*AGO2* rs11996715C>A		*AGO2* rs2292779C>G		*AGO2* rs4961280C>A		Database
		G allele	A allele	A allele	G allele	C allele	A allele	C allele	G allele	C allele	A allele	
African	661	0.884	0.116	0.512	0.488	0.817	0.183	0.916	0.084	0.967	0.033	1000Genome^1^
Ad Mixed American	347	0.467	0.533	0.386	0.614	0.562	0.438	0.614	0.386	0.725	0.275	
East Asian	504	0.775	0.225	0.656	0.344	0.587	0.413	0.605	0.395	0.881	0.119	
Chinese Dai in Xishuangbanna, China	93	0.737	0.263	0.581	0.419	0.527	0.473	0.586	0.414	0.876	0.124	
Han Chinese in Beijing, China	103	0.806	0.194	0.743	0.257	0.641	0.359	0.699	0.301	0.874	0.126	
Southern Han Chinese, China	105	0.810	0.190	0.695	0.305	0.567	0.433	0.610	0.390	0.881	0.119	
Japanese in Tokyo, Japan	104	0.755	0.245	0.654	0.346	0.591	0.409	0.538	0.462	0.904	0.096	
Kinh in Ho Chi Minh City, Vietnam	99	0.763	0.237	0.596	0.404	0.606	0.394	0.591	0.409	0.869	0.131	
European	503	0.141	0.859	0.088	0.912	0.459	0.541	0.494	0.506	0.831	0.169	
South Asian	489	0.327	0.673	0.137	0.863	0.540	0.460	0.624	0.376	0.773	0.227	
African	7625	0.784	0.216	0.454	0.546	0.753	0.247	0.873	0.127	0.946	0.053	gnomAD^2^
Ad Mixed American	16,574	0.458	0.542	0.385	0.615	0.580	0.420	0.671	0.329	0.693	0.307	
Ashkenazi Jewish	4654	0.219	0.781	0.159	0.841	0.407	0.593	0.528	0.472	0.821	0.179	
East Asian	8605	0.824	0.176	0.738	0.262	0.606	0.394	0.628	0.372	0.891	0.109	
Finnish	10,983	0.184	0.816	0.127	0.873	0.528	0.472	0.485	0.515	0.808	0.192	
Non-Finnish European	54,560	0.137	0.863	0.920	0.080	0.488	0.512	0.514	0.486	0.811	0.189	
South Asian	15,038	0.300	0.700	-	-	-	-	0.607	0.393	-	-	
Other (population not assigned)	2683	0.242	0.758	0.126	0.874	0.541	0.459	0.534	0.466	0.795	0.205	
Korean women (controls)	235	0.877	0.123	0.723	0.277	0.682	0.318	0.633	0.367	0.948	0.052	*Present study*
Korean women (RIF)	119	0.859	0.141	0.754	0.246	0.659	0.341	0.623	0.377	0.895	0.105	*Present study*
		[44]		[45–48]		[49]		[50]		[44]		References

1 The 1000 Genome Project website: https://www.internationalgenome.org/; we checked these polymorphisms frequencies based on GRCh38.

2 The Genome Aggregation Database website: http://gnomad.broadinstitute.org/; Official gnomAD release (version 2.0).

There were several limitations to the present study that should be considered when interpreting the results. First, it has remained unclear how these *AGO* polymorphisms might affect RIF. However, the studies of *AGO* polymorphisms and the expression difference in conformity with these polymorphisms have been elucidated in another animal and other conditions [35,52,58–61]. Second, a limited number of patients were included in our analyses. Therefore, future studies are needed to confirm that *AGO1* and *AGO2* play a critical role in RIF pathogenesis and to provide additional evidence that the regulation of *AGO1* and *AGO2* expression or activation can be used as a tool to prevent RIF. Nonetheless, our findings suggest that these polymorphisms may be potential biomarkers to diagnose and assess risk of RIF.

In conclusion, we identified associations between the *AGO2* rs4961280C>A polymorphism and prevalence of RIF in the Korean population, as well as a significant association between *AGO1* and *AGO2* gene polymorphisms and risk factors of RIF. However, the specific mechanisms underlying these effects require further investigation.

## Supplementary Material

Supplementary Tables S1-S4

## Abbreviations

AGO: argonaute
AOR: adjusted odds ratio
HWE: Hardy–Weinberg equilibrium
RIF: recurrent implantation failure
