# Supplementary material for: The association of AGO1 (rs595961G>A, rs636832A>G) and AGO2 (rs11996715C>A, rs2292779C>G, rs4961280C>A) polymorphisms and risk of recurrent implantation failure
Source: Biosci Rep. 2019 Nov 26;39(11):BSR20190342. doi: 10.1042/BSR20190342 (PMC6881209; doi:10.1042/BSR20190342)
Supplement: Supplementary Tables S1-S4 [file BSR-2019-0342_supp.pdf]

Supplementary Table 1. Information of *AGO1, AGO2* polymorphisms for PCR-RFLP and real-time PCR analysis.

Gene	Rs number	CHR	Position	Primer sequence	Probe sequence	Annealing temperature	Restriction enzyme	Genotypes size
AGO1	rs595961	ch1	35902179	F: 5'- CCC TAC ATC CAG GAA TTT GGG -3' R: 5'- TCG ACA CTG TTT TTG GGG TG -3'		58ºC	*Bfa*I	GG: 349bp GA: 349bp, 203bp, 146bp AA: 203bp, 146bp
AGO1	rs636832	ch1	35897874	F: 5'- CTG ATT CCA GAA CAT ATC ACT CAT -3' R: 5'- GGT ATA CCC AGA GAC TGA AAG TAA A -3'		55ºC	*Nla*III	GG: 96bp, 25bp GA: 121bp, 96bp, 25bp AA: 121bp
AGO2	rs11996715	ch8	140637192	F: 5'- GTT CCT TCC TGC AAT GGT GCC CTT -3' R: 5'- ATG TGA AGG AGA CAG GGG CAG AGA -3'	Oligo 5' - TCT GTA CCT TTA CTG TAG TAT TTC AAG GAA ATT TTT AAA TAA TCA TGA ATG - 3' Oligo 5' - TCT GTA CCT TTA CTG TAG TAT TTC ACG GAA ATT TTT AAA TAA TCA TGA ATG - 3'	60ºC		
AGO2	rs2292779	ch8	140551294	F: 5'- CGG AAC AAG CAG TTC CAC AC -3' R: 5'- TGA CAG GGA AAG GCT GAT GA -3'		58ºC	*Aci*I	CC: 142bp, 23bp CG: 165bp, 142bp, 23bp GG: 165bp
AGO2	rs4961280	ch8	140637315	F: 5’ - TGC CCC TGT CTC CTT CAC ATG TCC – 3’ R: 5’ - GTT CCC CAA CAC AGC GCT CAA AGG – 3’		58ºC	*Hpy*166II	CC: 169bp CA: 169bp, 146bp, 23bp AA: 146bp, 23bp

Note: PCR-RFLP, polymorphism chain reaction-restriction fragment length polymorphism; AGO, argonaute; CHR, chromosome; Rs number, references number.

Supplementary Table 2. Combined genotype analysis for the Argonaute gene polymorphisms in RIF patients and controls

Combined genotypes	Controls (n=211)	RIF patients (n=167)	AOR (95% CI)*	*P*
AGO1 rs595961G>A/*AGO1* rs636832A>G				
GG/AA	84 (39.8)	94 (56.3)	1.000 (reference)	
GG/AG	72 (34.1)	25 (15.0)	0.312 (0.181 - 0.538)	<0.0001
GG/GG	7 (3.3)	4 (2.4)	0.512 (0.145 - 1.810)	0.298
GA/AA	19 (9.0)	1 (0.6)	0.047 (0.006 - 0.358)	0.003
GA/AG	23 (10.9)	36 (21.6)	1.401 (0.766 - 2.562)	0.274
GA/GG	2 (0.9)	4 (2.4)	1.790 (0.319 - 10.050)	0.508
AA/AA	2 (0.9)	0 (0.0)	NA	0.998
AA/AG	0 (0.0)	1 (0.6)	NA	0.998
AA/GG	2 (0.9)	2 (1.2)	0.895 (0.123 - 6.494)	0.912
AGO1 rs595961G>A/*AGO2* rs4961280C>A				
GG/CC	147 (69.7)	100 (59.9)	1.000 (reference)	
GG/CA	16 (7.6)	22 (13.2)	2.013 (1.007 - 4.025)	0.048
GG/AA	0 (0.0)	1 (0.6)	NA	0.998
GA/CC	39 (18.5)	31 (18.6)	1.172 (0.685 - 2.004)	0.563
GA/CA	5 (2.4)	9 (5.4)	2.656 (0.864 - 8.164)	0.088
GA/AA	0 (0.0)	1 (0.6)	NA	0.998
AA/CC	3 (1.4)	3 (1.8)	1.468 (0.290 - 7.424)	0.643
AA/CA	1 (0.5)	0 (0.0)	NA	0.998
AA/AA	0 (0.0)	0 (0.0)	NA	NA
AGO2 rs11996715C>A/*AGO2* rs4961280C>A				
CC/CC	88 (41.7)	52 (31.1)	1.000 (reference)	
CC/CA	15 (7.1)	19 (11.4)	2.150 (1.005 - 4.601)	0.049
CC/AA	0 (0.0)	0 (0.0)	NA	NA
CA/CC	75 (35.5)	64 (38.3)	1.445 (0.894 - 2.337)	0.133
CA/CA	7 (3.3)	12 (7.2)	2.857 (1.056 - 7.729)	0.039
CA/AA	0 (0.0)	2 (1.2)	NA	0.998
AA/CC	26 (12.3)	18 (10.8)	1.165 (0.583 - 2.329)	0.666
AA/CA	0 (0.0)	0 (0.0)	NA	NA
AA/AA	0 (0.0)	0 (0.0)	NA	NA
AGO2 rs2292779C>G/*AGO2* rs4961280C>A				
CC/CC	74 (35.1)	53 (31.7)	1.000 (reference)	
CC/CA	5 (2.4)	15 (9.0)	4.225 (1.442 - 12.378)	0.009
CC/AA	0 (0.0)	1 (0.6)	NA	0.998
CG/CC	96 (45.5)	59 (35.3)	0.856 (0.529 - 1.384)	0.526
CG/CA	13 (6.2)	10 (6.0)	1.071 (0.437 - 2.628)	0.881
CG/AA	0 (0.0)	1 (0.6)	NA	0.998
GG/CC	19 (9.0)	22 (13.2)	1.629 (0.800 - 3.314)	0.178
GG/CA	4 (1.9)	6 (3.6)	2.102 (0.565 - 7.821)	0.268
GG/AA	0 (0.0)	0 (0.0)	NA	NA

RIF, recurrent implantation failure; AGO, argonaute; 95% CI, 95% confidence interval; AOR, adjusted odds ratio; N/A, not applicable. * Adjusted by age

Supplementary Table 3. Clinical variables in recurrent implantation failure patients stratified by *AGO1, AGO2* polymorphisms status by ANOVA

Genotypes	Homocysteine (mmol/L) Mean ± SD	P^*	WBC (10^3/uL) Mean ± SD	P^*	aPTT (sec) Mean ± SD	P^*	LH (mIU/mL) Mean ± SD	P^*
AGO1 rs595961G>A								
GG	6.76±1.42	0.946	7.49±2.92	0.265	29.75±3.36	0.003	5.10±2.55	0.098
GA	6.79±1.80		6.57±2.65		27.69±2.68		4.14±1.80	
AA	7.26±0.00		5.98±1.80		32.07±5.93		6.81±0.33	
Dominant (GG vs GA+AA)	6.83±1.72	0.885	6.53±2.59	0.108	28.06±3.19	0.011	4.32±1.87	0.130
Recessive (GG+GA vs AA)	7.26±0.00	0.742	5.98±1.80	0.536	32.07±5.93	0.156	6.81±0.33	0.251
AGO1 rs636832A>G								
AA	6.78±1.49	0.991	7.63±3.08	0.219	29.65±3.39	0.382	5.40±2.64	0.081†
AG	6.79±1.50		6.68±2.43		28.83±3.42		4.17±1.93	
GG	6.67±1.25		6.90±3.15		28.90±3.04		4.95±1.87	
Dominant (AA vs AG+GG)	6.77±1.43	0.993	6.70±2.46	0.082	28.83±3.35	0.165	4.27±1.92	0.037†
Recessive (AA+AG vs GG)	6.67±1.25	0.896	6.90±3.15	0.817	28.90±3.04	0.758	4.95±1.87	0.938
AGO2 rs11996715C>A								
CC	6.63±1.30	0.812	7.44±3.31	0.760	29.51±3.40	0.663	4.54±2.44	0.432
CA	6.89±1.68		7.04±2.38		29.33±3.62		5.18±2.42	
AA	6.81±1.16		7.04±2.79		28.63±2.25		4.94±2.02	
Dominant (CC vs CA+AA)	6.87±1.57	0.528	7.04±2.43	0.458	29.19±3.40	0.593	5.14±2.34	0.207
Recessive (CC+CA vs AA)	6.81±1.16	0.949	7.04±2.79	0.826	28.63±2.25	0.579†	4.94±2.02	0.931
AGO2 rs2292779C>G								
CC	6.58±1.36	0.715	6.52±2.55	0.044	29.59±3.55	0.644	4.51±2.04	0.244
CG	6.91±1.48		7.46±3.02		29.01±3.42		5.30±2.75	
GG	6.84±1.70		8.42±2.74		29.46±2.91		4.53±1.91	
Dominant (CC vs CG+GG)	6.89±1.52	0.416	7.70±2.97	0.029	29.13±3.28	0.432	5.14±2.60	0.183
Recessive (CC+CG vs GG)	6.84±1.70	0.870	8.42±2.74	0.060	29.46±2.91	0.841	4.53±1.91	0.583
AGO2 rs4961280C>A								
CC	6.94±1.46	0.039	7.00±2.79	0.280	29.39±3.32	0.684	5.02±2.49	0.505
CA	5.90±1.22		8.04±3.13		29.18±3.78		4.38±2.05	
AA	-		8.14±1.34		27.35±0.07		4.03±0.02	
Dominant (CC vs CA+AA)	5.90±1.22	0.039	8.05±3.01	0.110	29.06±3.68	0.638	4.35±1.95	0.249
Recessive (CC+CA vs AA)	-	-	8.14±1.34	0.648	27.35±0.07	0.410	4.03±0.02	0.613

ANOVA, analysis of variance; WBC, white blood cell; aPTT, activated partial thromboplastin time; LH, luteinizing hormone; SD, standard deviation.

*Calculated using ANOVA. †Calculated using the Kruskal-Wallis test.

Supplementary Table 4. Clinical variables in recurrent implantation failure patients stratified by *AGO1*, *AGO2* polymorphisms status by ANOVA

Genotypes	CD3$^+$ pan T cell (%)		CD4$^+$ helper T cell (%)		CD8$^+$ suppressor T cell (%)		CD19$^+$ B cell (%)	
	Mean ± SD	P^*	Mean ± SD	P^*	Mean ± SD	P^*	Mean ± SD	P^*
AGO1 rs595961G>A								
GG	68.39±8.52	0.313†	36.81±8.69	0.017	27.63±7.71	0.109	11.69±4.68	0.199
GA	65.08±15.26		31.84±9.22		31.03±8.17		10.09±3.77	
AA	51.00±0.00		24.00±0.00		23.00±0.00		8.00±0.00	
Dominant (GG vs GA+AA)	64.61±15.21	0.532†	31.58±9.17	0.006	30.76±8.16	0.064	10.02±3.72	0.082
Recessive (GG+GA vs AA)	51.00±0.00	0.127	24.00±0.00	0.209	23.00±0.00	0.490	8.00±0.00	0.470
AGO1 rs636832A>G								
AA	69.33±8.76	0.046	36.60±8.75	0.235	28.41±8.09	0.996	11.27±4.52	0.906
AG	64.39±12.35		33.65±9.28		28.51±7.58		11.32±4.05	
GG	70.61±12.06		36.84±10.03		28.64±9.82		10.51±7.15	
Dominant (AA vs AG+GG)	65.21±12.38	0.043	34.07±9.34	0.142	28.53±7.80	0.937	11.21±4.49	0.948
Recessive (AA+AG vs GG)	70.61±12.06	0.415	36.84±10.03	0.667	28.64±9.82	0.952	10.51±7.15	0.659
AGO2 rs11996715C>A								
CC	66.06±12.89	0.479	35.23±8.97	0.296	28.59±7.61	0.171	11.40±4.89	0.835
CA	68.64±8.17		34.65±9.04		29.33±8.44		10.96±3.89	
AA	68.08±10.10		39.20±9.59		24.53±6.32		11.68±5.22	
Dominant (CC vs CA+AA)	68.53±8.50	0.228	35.56±9.25	0.848	28.37±8.24	0.885	11.11±4.15	0.732
Recessive (CC+CA vs AA)	68.08±10.10	0.814	39.20±9.59	0.126	24.53±6.32	0.068	11.68±5.22	0.721
AGO2 rs2292779C>G								
CC	66.00±12.89	0.421	34.48±8.31	0.302	29.38±7.53	0.453	12.28±4.92	0.104
CG	67.85±9.40		35.11±8.74		28.27±7.99		10.46±3.94	
GG	69.58±7.66		38.11±11.19		26.79±8.69		10.46±4.21	
Dominant (CC vs CG+GG)	68.42±8.84	0.241	36.10±9.63	0.353	27.79±8.19	0.295	10.46±4.00	0.033
Recessive (CC+CG vs GG)	69.58±7.66	0.303	38.11±11.19	0.130	26.79±8.69	0.284	10.46±4.21	0.379
AGO2 rs4961280C>A								
CC	67.99±9.62	0.267	35.82±9.16	0.101	28.31±7.82	0.061	10.89±4.31	0.056
CA	64.51±14.51		35.02±8.38		27.95±7.76		12.97±4.88	
AA	74.00±1.41		22.00±4.24		41.50±4.95		7.00±1.41	
Dominant (CC vs CA+AA)	65.27±14.14	0.269	33.98±8.84	0.375	29.04±8.39	0.686	12.49±4.97	0.116
Recessive (CC+CA vs AA)	74.00±1.41	0.383	22.00±4.24	0.035	41.50±4.95	0.018	7.00±1.41	0.178

ANOVA, analysis of variance; SD, standard deviation. *Calculated using ANOVA. †Calculated using the Kruskal-Wallis test.
